# Prevalence of Periodontitis among Patients Diagnosed with Marfan Syndrome: A Cross-Sectional Study Comparing Samples of Healthy Patients

**DOI:** 10.1155/2022/6238099

**Published:** 2022-06-01

**Authors:** Cosimo Galletti, Jorge Toledano-Serrabona, Octavi Camps-Font, Gisela Teixido-Tura, Inmaculada Llobet-Poal, Carles Subirà-Pifarré, Luca Fiorillo, Cosme Gay-Escoda

**Affiliations:** ^1^Faculty of Medicine and Health Sciences, School of Dentistry, University of Barcelona, IDIBELL (Bellvitge Biomedical Research Institute), 08907 Barcelona, Spain; ^2^Department of Cardiology, Hospital Universitari Vall d'Hebron, Autonomous University of Barcelona, 08035 Barcelona, Spain; ^3^Faculty of Medicine and Health Sciences, School of Dentistry, University of Barcelona, 08907 Barcelona, Spain; ^4^Multidisciplinary Department of Medical-Surgical and Odontostomatological Specialties, University of Campania “Luigi Vanvitelli”, 80121 Naples, Italy; ^5^School of Dentistry Department of Biomedical and Dental Sciences and Morphofunctional Imaging, Uni-versity of Messina, via Consolare Valeria, 1, 98125 Messina, Italy; ^6^Department of Dentistry, University of Aldent, 1000 Tirana, Albania; ^7^Oral and Maxillofacial Surgery, Faculty of Medicine and Health Science, School of Dentistry, University of Barcelona, Barcelona, Spain; ^8^Oral Surgery and Implantology (EHFRE International University/FUCSO), Bellvitge Biomedical Research Institute (IDIBELL), 08907 Barcelona, Spain

## Abstract

Periodontitis is an inflammatory condition caused by a bacterial plaque and characterized by progressive destruction of the tooth-supporting apparatus. Patients with Marfan syndrome (MFS) exhibit a connective tissue disorder, which can also affect oral soft and hard tissue. Thus, the aims of this cross-sectional study were to assess the association between periodontitis and MFS and secondly, to compare periodontal parameters and prevalence of disease with a control group (CG) without MFS. 152 patients (MFS = 76, CG = 76) were recruited to evaluate the following periodontal parameters: probing depth, gingival margin, clinical attachment level, plaque index, and bleeding on probing. The 2017 World Workshop guideline was followed for the diagnosis of the periodontal status. A multivariate analysis was performed using a multinomial logistic regression adjusted for age, gender, and smoking. The level of significance required was *p* < 0.05. Patients with MFS did not show a higher prevalence of periodontitis compared to the CG. However, patients with MFS did have higher values in probing depth, gingival recession, clinical attachment level, and plaque index compared to the CG patients (*p* < 0.05). In conclusion, although similar prevalence of periodontitis was found among the studied groups, MFS patients showed worse periodontal parameters.

## 1. Introduction

Periodontal disease is a multifactorial chronic inflammatory disease. In an initial phase, a dysbiotic dental plaque initiates gingival inflammation. In susceptible patients, gingivitis could progress to periodontitis due to changes in the bacterial clusters, if left untreated [1]. Periodontitis is characterized by the progressive destruction of the supporting apparatus of the tooth. In general, patients with periodontal disease show gingival inflammation and a worsening of periodontal clinical parameters such as bleeding on probing, increased periodontal probing depth, gingival recession with root exposure, or even tooth loss [2].

It has been suggested that there is a relationship between several systemic diseases and periodontitis due to a direct transmission of bacteria, generated by ulceration of the periodontal pocket epithelium, to the systemic bloodstream, or due to indirect systemic effects caused by an inflammatory condition, generated by the presence of periodontal bacteria and their products, such as endotoxin or lipopolysaccharide [3–5]. Therefore, patients with periodontitis often present elevated levels of C-reactive protein (CRP), which is a parameter used to determine systemic inflammation [6, 7].

On the other hand, systemic disorders, such as uncontrolled diabetes mellitus, neoplastic disease, or Papillon Lefevre syndrome, may also affect the periodontal tissues modifying the severity of periodontal disease [8]. In the past few years, it has been pointed out that there is a possible influence of the genetic disease Marfan syndrome (MFS) on the development of periodontal diseases [9]. MFS is a multisystem connective tissue disorder consisting of a defect in the microfibrillar protein fibrillin-1 gene (FBN1) on chromosome 15 (15q21.1). FBN1 monomers bond to form complex extracellular macroaggregates, called microfibrils, which form part of elastic fibers, and confer important biomechanical properties in connecting, anchoring, and maintaining tissues and organs [10]. In addition, it has been proven that FBN1 stimulates the release and activation of the Transforming Growth Factor (TGF), a potent inflammation stimulator that promotes fibrosis and activates certain matrix metalloproteinases (MMPs), especially MMP-2 and MMP-9 [11].

MFS can affect the cardiovascular, skeletal, and ocular systems, with aortic dissection being the major cause of mortality. Furthermore, patients with MFS can also exhibit oral manifestations including retrognathia, dolichocephaly, high palatal vault, crowded teeth, temporomandibular joint disorders, and partial anodontia [9, 12]. Taking into consideration that periodontal ligament is mainly made of connective tissue, a higher incidence of periodontal diseases can be expected among patients with MFS [13, 14].

Several studies have also demonstrated that there is an association between periodontitis and coronary heart disease [15, 16]. In fact, a recent workshop of the American Academy of Periodontology (AAP) and the European Federation of Periodontology (EFP) [17] established that periodontitis represents a risk factor for the development of cardiovascular disease, so dentists should inform their patients, especially those at risk.

Patients with MFS are often heart valve carriers and have an increased risk of cardiovascular complications. Thus, these patients must maintain good oral health, preventing the bacteraemia that could be caused by advanced tooth decay, pulpal infection, and/or periodontal diseases [18]. The diagnosis of MFS is challenging since many of its manifestations are present in other syndromes as well as in the general population. Although genetic tests are available, the diagnostic criteria of the current Ghent nosology still require clinical manifestations for final diagnosis [19].

It is therefore important to determine the association between periodontal diseases and the presence of MFS in order to establish a strict periodontal maintenance program among these patients. However, there are few studies that compare the prevalence of periodontal disease in MFS patients and these show contradictory results [9, 12, 20].

The main aim of this cross-sectional study was to assess the association between MFS and periodontitis. The second objective was to compare the prevalence of periodontal diseases and periodontal parameters with a control group of patients without MFS.

## 2. Materials and Methods

### 2.1. Study Design, Setting, and Participants

A cross-sectional study was conducted in accordance with the Strengthening the Reporting of Observational studies in Epidemiology (STROBE) guidelines for conducting cross-sectional studies [21]. The protocol complied with the Helsinki Declaration and was approved by the Ethical Committee for Clinical Research (CEIC) of the Dental Hospital of Bellvitge of the University of Barcelona (Spain) under number 34/2019 and of the Vall d'Hebron University Hospital of Barcelona (Spain) under number 378/2017.

The MFS group included individuals who met the following criteria: (1) patients with a confirmed diagnosis of MFS through clinical and genetic testing, (2) those who were between 20 and 80 years of age, and (3) those who consecutively visited the Marfan Service located inside the Cardiology Department of the Vall d'Hebron Hospital Campus (Barcelona, Spain), regardless of the presence or absence of cardiovascular disease (CVD). Patients with systemic disorders different from MFS, patients who had taken anti-inflammatories or antibiotics in the past 14 days, and pregnant patients were excluded, being potentially more prone to changes in gingival inflammatory status. The control group (CG) was composed of patients that (1) had no diagnosis of MFS or any other systemic disorder, (2) were between 20 and 80 years old, and (3) consecutively visited the University of Barcelona Dental Hospital. Just as the MFS group, patients who had taken anti-inflammatories or antibiotics in the past 14 days and pregnant patients were also excluded from the control group.

Before the recruitment, information about the study design and the objective of the paper was explained to the patients. All patients signed an informed consent form before recruitment.

### 2.2. Variables and Measurement

All patients underwent a comprehensive periodontal examination. All dental examinations were carried out by a single, experienced, and calibrated investigator (C.G.), which always used the same periodontogram chart from the Spanish Society of Periodontology and Osteointegration (SEPA).

The following variables were recorded:

- Probing depth (PD): distance from the gingival margin to the tip of the periodontal probe, measured at 6 points for each tooth (buccal: mesial, medial, and distal; palatine/lingual: mesial, medial, and distal). Expressed in millimeters
- Gingival margin (GM): distance from the gingival margin to the cementoenamel junction, measured at 6 points for each tooth (buccal: mesial, medial, and distal; palatine/lingual: mesial, medial, and distal), expressed in millimeters
- Clinical attachment level (CAL): probing depth plus or minus the gingival margin, measured at 6 points for each tooth (buccal: mesial, medial, and distal; palatine/lingual: mesial, medial, and distal), expressed in millimeters
- O'Leary plaque index (PI): presence of plaque at the dentogingival junction, measured at 6 points for each tooth (buccal: mesial, medial, and distal; palatine/lingual: mesial, medial, and distal), expressed as the percentage (%) of gingival surfaces with the presence of plaque
- Bleeding on probing (BOP): visual observation of bleeding through the gingival sulcus when determining probing depth, measured at 6 points for each tooth (buccal: mesial, medial, and distal; palatine/lingual: mesial, medial, and distal), expressed as the percentage (%) of gingival surfaces with the presence of bleeding on probing; calculated by dividing the number of sites where bleeding occurred by the total number of available sites in the mouth and multiplied by 100
- Suppuration on probing (SOP): visual observation of suppuration through the gingival sulcus when determining probing depth, measured at 6 points for each tooth (buccal: mesial, medial, and distal; palatine/lingual: mesial, medial, and distal), expressed as the percentage (%) of gingival surfaces with the presence of suppuration on probing; calculated by dividing the number of sites where suppuration is recorded is by the total number of available sites in the mouth and multiplied by 100
- Missing teeth: lack of teeth through disease or extraction, expressed as absolute values

In addition, variables such as gender, age, and smoking habits were also recorded for each patient. The measurements were made using a pressure-calibrated probe (Florida Probe®, Florida Probe Corporation, Gainesville, USA). The hemispheric probe tip had a diameter of 0.45 mm and a constant probing force of 15 g.

Diagnosis of periodontal status was based on epidemiological studies [22, 23]:

- Periodontal health was defined as an intact periodontium or a reduced and stable periodontium with <10% bleeding on probing sites and probing depth ≤ 3 mm
- Gingivitis was defined as an inflammatory lesion induced by bacterial plaque that remains confined to the gingival level and does not extend to the periodontal attachment; it is reversible and does not involve periodontal bone loss, with ≥10% bleeding on probing sites and probing depth ≤ 3 mm
- Periodontitis was diagnosed in individuals with CAL ≥ 3 mm and probing depth ≥ 3 mm at ≥2 nonadjacent teeth, not ascribable to nonperiodontitis-related causes (traumatic origin, caries extension, molar extraction etc.), and >10% bleeding on probing sites [24]

To test intraexaminer agreement and consistency, 10 patients were randomly reassessed after 15 days, showing excellent reliability (intraclass correlation coefficient, *r* = 0.91) and consistency (*r* = 0.97).

### 2.3. Sample Size Calculation

The sample size calculation was based on the assumptions that 16% of the Spanish population between 20 and 44 years of age suffer from periodontitis [25] and that suffering from MFS increases the risk of periodontitis at least 2.5-fold. Considering an *α* risk of 0.05, a power of 90%, and a 10% exclusion rate, 76 patients with MFS were required. One control, matched for sex and age (maximum difference ± 5 years), was selected for every case.

### 2.4. Statistical Analysis

Logic, range, and consistency tests of the results to debug the data. Additionally, the distribution of missing values and study variables were analyzed.

The subjects' characteristics were presented as absolute and relative frequencies for categorical outcomes. Normality of scale variables (patient age, PD, GM, CAL, PI, BOP, SOP, and number of missing teeth) was explored using the Shapiro-Wilks test and through the visual analysis of the P-P plot and a box plot. If the distribution was compatible with normality, the mean and standard deviation (SD) were used.

The association between periodontal status and MFS was assessed by means of multinomial logistic regression and reporting Odds Ratios (OR) and their 95% confidence intervals (95% CI), before and after adjusting for age, gender, and smoking habit. Linear regression analyses were performed to compare each of the clinical parameters assessed (PD, GM, CAL, PI, BOP, SOP, and number of missing teeth) between groups, reporting their mean difference (MD) and 95% CI before and after adjusting for the mentioned covariates. Additionally, we performed a subanalysis of patients with Marfan syndrome with CVD in order to analyze its effect on periodontal parameters.

The data was analyzed using Stata14 (StataCorp., College Station, USA) software by an independent investigator (OC-F). In all cases, the statistical significance level was set at a *p* value < 0.05.

## 3. Results

### 3.1. Participants

The MFS and CG is comprised of 76 patients (30 males and 46 females) each. There were no statistical differences between groups in terms of age (*p* value = 0.533) and smoking (*p* value = 0.368). The clinical characteristics of the participants are summarized in Table 1.

### 3.2. Main Results

The crude risk of periodontitis was almost twice in patients with Marfan (OR = 1.81, 95% CI: 0.80 to 4.09) but not statistically significant (*p* > 0.05). After adjusting for age, gender, and smoking habit, the risk of periodontitis did not change substantially (OR = 2.06, 95% CI: 0.87 to 4.89) with the remaining being not significant (Table 2).

Mean and SD values for all periodontal parameters (PD, GM, CAL, BOP, PI, and number of missing teeth) are presented in Table 3. MFS patients were associated with significantly higher levels of periodontal probing depth (2.83 mm, SD = 0.43 mm vs. 2.53 mm, SD = 0.61 mm; MD = 0.31 mm, 95% CI: 0.15 to 0.47 mm; *p* value < 0.001), gingival recession (0.41 mm, SD = 0.79 mm vs. 0.22 mm, SD = 0.60 mm; MD = 0.21 mm, 95% CI: 0.003 to 0.41 mm; *p* value < 0.005), clinical attachment level (3.24 mm, SD = 0.98 mm vs. 2.74 mm, SD = 0.91 mm; MD = 0.52 mm, 95% CI: 0.26 to 0.79 mm; *p* value < 0.001), and plaque index (50.4%, SD = 31.5% vs. 39.7%, SD = 30.4%; MD = 11.7%, 95% CI: 2.01 to 21.5%; *p* value < 0.005). No statistically significant differences were found between the MFS and the CG in terms of bleeding on probing (18.7%, SD = 19.7% vs. 16.6%, SD = 17.2%; MD = 2.5%, 95% CI: -3.3 to 8.4%; *p* value > 0.005) and missing teeth (5.5, SD = 4.6 vs. 6.3 mm, SD = 4.7; MD = −0.72, 95% CI: -2.02 to 0.58; *p* value > 0.005). None of the groups presented SOP. The assumptions of the models were fulfilled for each outcome variable.

Periodontal parameters of patients with Marfan syndrome were not influenced by the presence or absence of cardiovascular disease (Table 4).

## 4. Discussion

MFS is a connective tissue disease characterized by a series of systemic alterations [9, 12]. Specifically, MFS is caused by a mutation of the FBN1 gene, which translates into fibrillin-1, an extracellular matrix protein important in elastic and non-elastic connective tissues. Signs or symptoms of this condition depend on which system is affected and the severity. MFS most commonly affect patients by the connective tissue of the bones, eyes, heart and vessels, lungs, skin, and spinal cord. Cardiovascular alterations such as aortic dissection or ruptures are life-threating complications that increase the morbidity and mortality among these patients [26]. Besides the aforementioned systemic manifestations, the oral cavity can also be affected by dental crowding, narrow palate, temporomandibular disorders, and high palatine vault among the most common alterations [27–29]. Moreover, it has been hypothesized a higher prevalence of periodontitis among MFS patients occurs due to the high presence of connective tissue at the periodontal level [14]. In this sense, Shiga et al. [14] reported that the periodontal ligament of patients with MFS had higher levels of inflammatory cytokines such as TGF-*β*. Besides, fibrillin-1 has been suggested to be essential for normal tissue structure and gene expression of the periodontal ligament [30]. What is more, a recent study revealed higher MMP-13 activity in the crevicular fluid of MFS patients compared to healthy individuals, which could lead to significant degradation of the periodontal matrix and an altered response in case of periodontal inflammation [31].

The present cross-sectional study compared the prevalence of periodontal diseases in patients with MFS compared to a CG. The main periodontal parameters such as PPD, GR, CAL, BOP, SOP, PI, and missing teeth were also evaluated. Both groups had similar periodontal status, with similar prevalence of periodontal diseases (gingivitis and periodontitis). However, multivariate analysis showed that the cohort of patients with MFS had higher values of PPD, GR, CAL, and plaque index. Unfortunately, due to the low prevalence of periodontitis in the present study, the statistical analysis could not be performed taking into account the severity of the disease. It is therefore recommended that future studies with larger sample sizes should address not only the influence of MFS on the risk of developing periodontal diseases but also the severity of clinical parameters.

The prevalence of periodontitis among MFS patients was 28.9%, while in the control group it was 22.4%. No statistically significant differences were found between groups in the prevalence of periodontal health, gingivitis, and periodontitis. These results do not agree with those previously published by Suzuki et al. [20] who reported that MFS patients had more periodontitis than patients without MFS. It should be noted that they carried out a different periodontal measurement (recording BoP and PD only on the four first molars and two central incisors) and diagnosed periodontitis on the basis of the community periodontal index (CPI). In our study, a more complete periodontal measurement was performed on all teeth in the mouth, and the periodontitis was diagnosed using the most recent criteria. Another difference could lie in the use of the Florida probe, which, compared to the manual probe used in Suzuki's study, could generate false negatives [32]. Besides, they recruited only 54 patients (40 MFS vs. 14 CG patients), and nearly all patients had cardiovascular complications, while in our study the sample of patients was randomly selected and consisted in patients with or without on-going cardiovascular complications. In fact, in another investigation, in which 47 patients with MFS with cardiovascular disease (CVD) and 48 without MFS with CVD were compared, a higher prevalence of periodontitis was found in patients with MFS with CVD [33]. Surprisingly, probably due to the small number of MFS patients with CVD, this association was not found in the present study (Table 4). Accordingly, future studies are needed to clarify the influence of CVD on the risk of developing periodontal disease in Marfan patients.

Regarding periodontal parameters, we found higher levels of PPD, GR, and CAL in MFS with respect to CG patients. BoP was comparable between case and controls, suggesting the fact that the syndrome does not seem to imply a worse gingival inflammation. Similar values of PD, GR, CAL, and BoP were found in the study of Staufenbiel et al. [9]. Besides, higher levels of PI were found in patients with MFS compared to CG. This fact could be explained due to the anatomical oral conditions of MFS patients (e.g., dental crowding, narrow palate, and high palatine vault), which may lead to a higher plaque accumulation with consequent gingival inflammation [9, 12]. Thus, the absence of a control over time of the patients does not allow to exclude the hypothesis that the greater plaque found in the patients at the time of the study may in the future generate greater gingival inflammation. Since periodontal inflammation worsens the prognosis of cardiovascular disease [17, 34, 35], it is important for MFS patients to maintain close control of oral hygiene levels and to incorporate these patients into a strict periodontal maintenance program.

One of the main limitations of the present study was the age of the recruited patients (44.6 and 44.8 years for MFS and CG, respectively). Carasol et al. [36] reported that the prevalence of periodontitis significantly increases in patients over 45 years of age. Besides, the manifestations of the MFS usually worsen over the years. Thus, it could be interesting to perform future studies with older patients. However, it must be pointed out that there were no statistical differences between the groups in terms of age, which means that both groups were equally affected by this confounding factor. In our opinion, our results may help to understand the possible association between MFS and periodontal diseases. The second limitation was related to the design of the present study; although cross-sectional studies are useful to study the prevalence of a disease, more studies with different designs are needed to assess the incidence or risk factors of periodontal disease among these sample of patients, taking into consideration other confounding factors (i.e., diabetes, immunodeficiency disorders, and use of certain drugs) which could predispose patients, with or without MFS, to periodontal disease. Moreover, collecting information on smoking in quantitative terms rather than as a binary variable could lead to more robust results in future studies [37]. In addition, another possible drawback of this study is related to the fact that patients were enrolled from different health centers: patients with MFS from Hospital Vall d'Hebron and patients of CG from Hospital de Bellvitge. Therefore, some confounding factors such as lifestyle, education, and socioeconomic aspects could also influence periodontal parameters [38–44].

## 5. Conclusions

Despite the limitations of the present study, prevalence of periodontal diseases among Marfan patients was similar compared to that among patients without this syndrome. On the other hand, patients with Marfan syndrome had worse periodontal probing depth, gingival recession, clinical attachment level, and plaque index.

Future studies controlling for patient confounders are needed in order to confirm the association between periodontal diseases and Marfan syndrome.

## Figures and Tables

**Table 1:** General characteristics of the study population.

	Marfan syndrome group (*n* = 76)	Control group (*n* = 76)	*p* value
Age (years), mean (SD)	44.6 (13.9)	44.8 (19.9)	0.533^**+**^
Smoking status, *N* (%)			
No smoker	57 (75.0)	52 (68.4)	0.368^**++**^
Smoker	19 (25.0)	24 (31.6)	

SD: standard deviation; *N*: number of observations. ^**+**^Paired *t*-test. ^**++**^McNemar *χ*^2^ test.

**Table 2:** Prevalence of periodontal health, gingivitis and periodontitis.

	Marfan syndrome group (*n* = 76)	Control group (*n* = 76)	OR (95% CI)^+^	OR (95% CI)^++^
Periodontal health, *N* (%)	25 (32.9)	35 (46.0)	1	1
Gingivitis, *N* (%)	29 (38.2)	24 (31.6)	1.69 (0.80 to 3.56)	1.78 (0.83 to 3.80)
Periodontitis, *N* (%)	22 (28.9)	17 (22.4)	1.81 (0.80 to 4.09)	2.06 (0.87 to 4.89)

CI: confidence interval; *N*: number of observations; OR: Odds Ratio. ^**+**^Simple multinomial logistic regression. ^**++**^Multivariate multinomial logistic regression adjusted for age, gender, and smoking.

**Table 3:** Mean and SD values of periodontal parameters and comparison of the mean differences.

	Marfan syndrome group (*n* = 76) Mean (SD)	Control group (*n* = 76) Mean (SD)	MD (95% CI)^+^	MD (95% CI)^++^
Probing depth (mm)	2.83 (0.43)	2.53 (0.61)	0.30 (0.13 to 0.47)^∗^	0.31 (0.15 to 0.47)^∗∗^
Gingival recession (mm)	0.41 (0.79)	0.22 (0.60)	0.19 (-0.03 to 0.42)	0.21 (0.003 to 0.41)^∗^
Clinical attachment level (mm)	3.24 (0.98)	2.74 (0.91)	0.5 (0.19 to 0.80)^∗^	0.52 (0.26 to 0.79)^∗∗^
Bleeding on probing (%)	18.7 (19.7)	16.6 (17.2)	2.2 (-3.7 to 8.1)	2.5 (-3.3 to 8.4)
Plaque index (%)	50.4 (31.5)	39.7 (30.4)	10.6 (0.7 to 20.6)^∗^	11.7 (2.01 to 21.5)^∗^
Missing teeth	5.5 (4.6)	6.3 (4.7)	-0.81 (-2.3 to 0.67)	-0.72 (-2.02 to 0.58)

CI: confidence interval; *N*: number of observations; MD: mean difference; ^∗^*p* < 0.05 and^∗∗^*p* < 0.001. ^**+**^Simple linear regression. ^**++**^Multivariate linear regression adjusted for age, gender, and smoking.

**Table 4:** Subanalysis of patients with Marfan syndrome with or without cardiovascular disease (CVD).

	Marfan syndrome group without CVD (*N* = 53) Mean (SD)	Marfan syndrome group with CVD (*N* = 23) Mean (SD)	MD (95% CI)^+^
Probing depth (mm)	2.83 (0.42)	2.84 (0.45)	0.005 (-0.2 to 0.21)
Gingival recession (mm)	0.45 (0.89)	0.31 (0.48)	-0.13 (-0.47 to 0.20)
Clinical attachment level (mm)	3.28 (1.06)	3.15 (0.75)	-0.12 (-0.54 to 0.28)
Bleeding on probing (%)	20.0 (20.9)	15.8 (16.8)	-3.1 (-13.0 to 6.9)
Plaque index (%)	53.6 (32.1)	43.1 (29.5)	-8.9 (-24.5 to 6.7)
Missing teeth	5.75 (5.24)	4.87 (2.76)	-0.54 (-2.60 to 1.51)

CI: confidence interval; *N*: number of observations; MD: mean difference. ^**+**^Multivariate linear regression adjusted for age, gender, and smoking.

## Data Availability

Data is available on request to authors.
